# Dynamic remodeling of portal vessels by Clec4g^+^ endothelial cells in liver growth and homeostasis

**DOI:** 10.1172/jci.insight.201391

**Published:** 2026-05-19

**Authors:** Sarah Platt, Norhan B.B. Mohammed, Joseph Brancale, Caroline Tippett, Kevin Seo, Sílvia Vilarinho

**Affiliations:** Departments of Medicine, Genetics, and Pathology, Yale University, New Haven, Connecticut, USA.

**Keywords:** Hepatology, Vascular biology, Endothelial cells, Mouse models

## Abstract

We generated an inducible mouse model to label and trace liver sinusoidal endothelial cells, revealing that Clec4g^+^ liver endothelial cells contribute to portal branches over time.

**To the Editor:** Liver endothelium, long overshadowed by hepatocytes, is now recognized as a central regulator of hepatic homeostasis (1). Liver sinusoidal endothelial cells (LSECs) constitute the liver’s specialized capillaries and the dominant endothelial subpopulation in a healthy liver, distinguished by the presence of fenestrations and lack of a basement membrane (2). LSECs regulate liver zonation and regeneration via angiocrine Wnt and HGF signaling (3). These pathways become dysregulated in many liver diseases, including cancer (2). Despite their essential role in maintaining hepatic homeostasis, LSECs remain challenging to isolate and study, leaving their in vivo dynamics largely unexplored (4). To address this gap, we first examined publicly available human and mouse single-cell RNA-seq datasets (Supplemental Figure 1A; supplemental material available online with this article; https://doi.org/10.1172/jci.insight.201391DS1), which confirmed that *Clec4g* is highly and selectively expressed in LSECs, supporting its suitability as a marker for in vivo labeling and tracing of these cells. Hence, we generated a *Clec4g*-CreERT2^+^ mouse line and crossed it with ZsGreen-Ai6^+^ reporter mice (Figure 1A). Tamoxifen-induced recombination at weaning age (3 weeks old) resulted in robust ZsGreen labeling of LSECs, as confirmed by confocal microscopy showing primary localization within hepatic sinusoids with rare positive cells at the sinusoidal interface of portal and central vessels (Supplemental Figure 1B). Among the organs examined, only the spleen showed a few ZsGreen^+^CD31^+^ endothelial cells (ECs) (Supplemental Figure 1C). Importantly, hepatic arteries and lymphatic vessels were spared of labeling, even 1 month after induction (Figure 1B). Flow cytometry confirmed efficient labeling of LSECs, with over two-thirds of liver ECs (CD45^–^CD31^+^) being ZsGreen^+^, while immune cells (CD45^+^CD31^–^) remained unlabeled (Figure 1C). Thus, the *Clec4g*-CreERT2^+^ × ZsGreen-Ai6^+^ mouse model is a powerful tool for efficient and selective in vivo labeling of LSECs, facilitating longitudinal studies of their dynamics over time.

At 7 days after tamoxifen, ZsGreen^+^ cells were largely confined to hepatic sinusoids, with minor presence in portal veins (PVs; surrounding bile ducts marked by E-cadherin) and central veins (CVs; surrounding zone 3 hepatocytes marked by Cyp2e1) (Figure 1, D and E). Longitudinal imaging from 7 days to 3 months revealed a progressive expansion of labeled cells from sinusoids into PVs. A modest expansion was also noted in CVs. These observations underscore an unanticipated contribution of Clec4g^+^ ECs to macrovascular venous remodeling (Figure 1, D and E).

Given the broad variability in the percentage of ZsGreen^+^ area across PV linings, vessels were stratified by cross-sectional area: small (≤572.1 μm²), intermediate (572.1–1248 μm²), and large (≥1,248 μm²). Two-way ANOVA revealed significant effects of PV size, postlabeling time, and their interaction on ZsGreen^+^ area (*P* < 0.001; Figure 1F). At 7 days, no significant differences were detected among the 3 size categories. At both 1 and 3 months, small PVs exhibited a higher proportion of ZsGreen^+^ cells than intermediate and large PVs, following a consistent size-dependent pattern (small > intermediate > large) (Figure 1F). Microscopy analyses further supported this observation spatially, showing preferential accumulation of Clec4g^+^ZsGreen^+^ cells within small PVs and terminal branches of portal venules over time (Figure 1G). To distinguish increased cell number from cell hypertrophy, we quantified DAPI^+^ nuclei within ZsGreen^+^ macrovascular regions and observed increased nuclei number over time (Figure 1H), indicating increased cell number.

To assess whether similar remodeling occurs in adult livers, lineage tracing of Clec4g^+^ ECs in 8-week-old *Clec4g*-CreERT2^+^ × ZsGreen-Ai6^+^ mice revealed that labeled cells, initially localized within the sinusoids, preferentially expanded into adjacent small PVs and, to a lesser extent, into CVs, over time (Supplemental Figure 2, A–E), mirroring postweaning patterns. See Supplemental Methods for methodological details (supplemental material available online with this article; https://doi.org/10.1172/jci.insight.201391DS1).

Collectively, these findings uncover a progressive, size-dependent enrichment of Clec4g^+^ZsGreen-Ai6^+^ cells within portal vessels, with small and terminal portal branches near the sinusoids representing the primary sites of repopulation. This pattern suggests that LSECs contribute to portal vessel remodeling in a retrograde manner — against the direction of blood flow — under homeostatic conditions (5). While most Clec4g^+^ cells in the liver are LSECs, it remains possible that proliferation of very rare Clec4g-expressing macrovascular ECs contributes to this process.

Our study has key implications. First, unlike the constitutive *Clec4g*-Cre model (6), this inducible system enables temporally controlled and selective lineage tracing of LSECs, with minimal off-target effects, providing a robust platform to investigate liver vascular biology in health and disease. Second, our data reveal a previously unrecognized role for Clec4g^+^ ECs in remodeling the portal vasculature, with distinct dynamics in small versus large vessels. Lastly, these findings reveal unanticipated aspects of liver EC behavior, offering potentially new perspectives on disease mechanisms and potential therapeutic strategies for endothelial-related liver disorders.

## Conflict of interest

SV serves as a consultant to Ipsen and Mirum Pharma and received research grant funding support from Moderna Therapeutics and Mirum.

## Funding support

The content is solely the responsibility of the authors and does not necessarily represent the official views of the NIH. This work is subject to the NIH Public Access Policy.

- National Institute of Diabetes and Digestive and Kidney Diseases of the National Institutes of Health (NIH) under award R01DK131033 (to SV), 5T32DK007017 (to NM), F30DK138640 (to JB), Microscopy Core - Yale Liver Center (P30DK034989).

## Supplementary Material

Supplemental data

Supporting data values

## Figures and Tables

**Figure 1:**
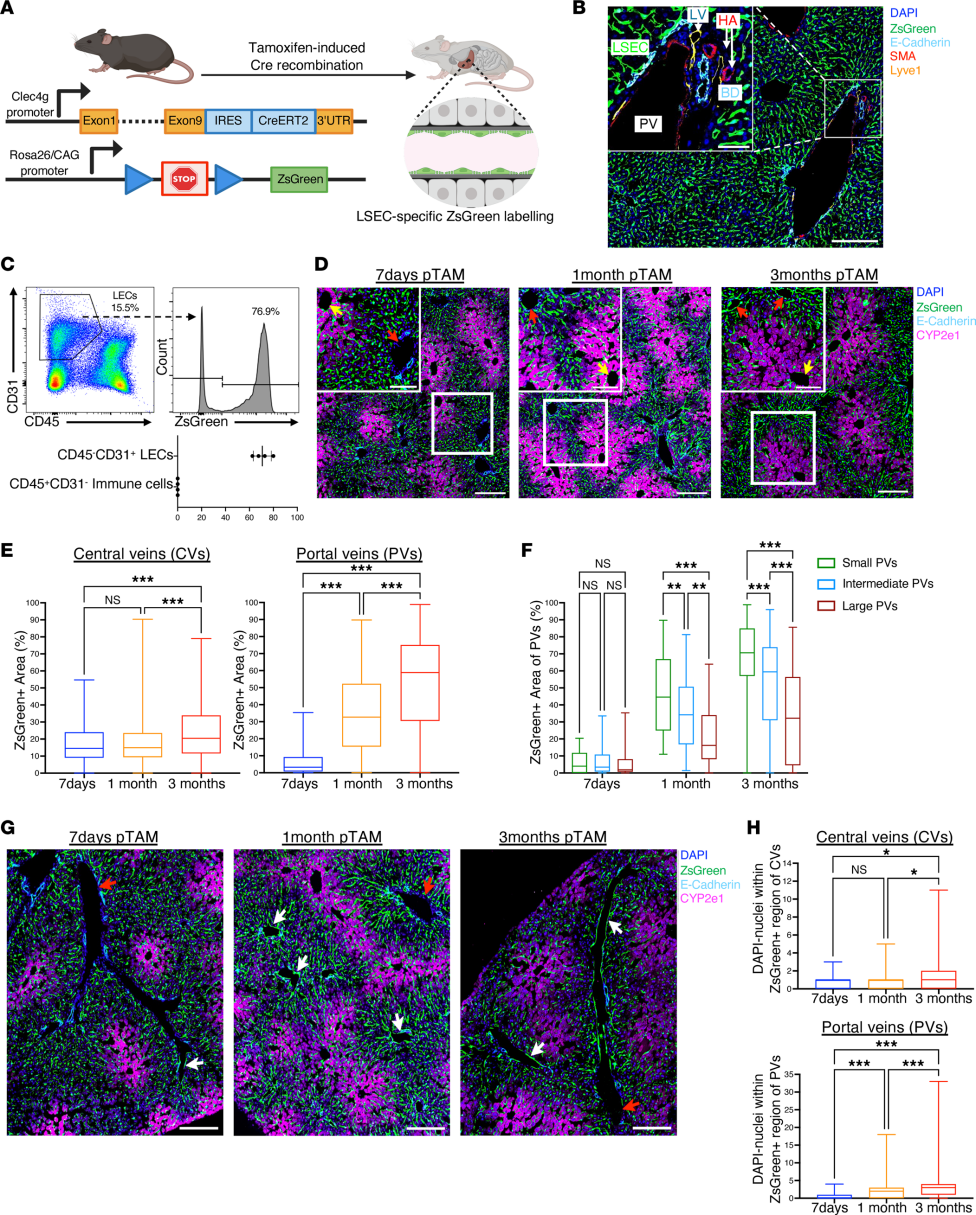
Progressive expansion of Clec4g^+^ endothelial cells into portal vessels following labeling at weaning age (3 weeks old). (**A**) Schematic representation of the *Clec4g*-CreERT2^+^ construct and ZsGreen-Ai6 reporter system. (**B**) Representative confocal image of a portal triad depicting a portal vein (PV), hepatic artery (HA), bile duct (BD), liver sinusoidal endothelial cells (LSECs), and lymphatic vessel (LV) 1 month after tamoxifen administration. Scale bars: 200 μm; 50 μm in insets. (**C**) Representative analysis of flow cytometry of CD45^–^CD31^+^ liver endothelial cells (LECs) showing ZsGreen^+^ and ZsGreen^–^ fractions, 7 days after tamoxifen administration (Top). Quantification of ZsGreen^+^ cells among CD45^–^CD31^+^ liver endothelial cells and CD45^+^CD31^–^ immune cells by flow cytometry (bottom; paired *t* test); *n* = 4 mice. (**D**) Representative confocal images at 7 days, 1 month, and 3 months after tamoxifen administration at weaning age. Scale bars: 200 μm; 100 μm in insets. PVs (red arrows) and CVs (yellow arrows). (**E**) Quantification of ZsGreen^+^ area in CVs and PVs over time (Kruskal-Wallis test); *n* = 4 mice per time point. (**F**) Quantification of ZsGreen^+^ area in PVs stratified by size (small, intermediate, large) at the indicated time points (2-way ANOVA); *n* = 4 mice per time point. (**G**) Representative confocal images of large PVs (red arrows) branching into terminal venules (white arrows), showing progressive accumulation of Clec4g^+^ZsGreen^+^ cells over time. Scale bars: 200 μm. (**H**) Quantification of DAPI^+^ nuclei within ZsGreen^+^ regions of CVs and PVs over time (Kruskal-Wallis test). SMA, smooth muscle actin; Lyve1, lymphatic vessel endothelial hyaluronan receptor 1. Scatter dot plot showing individual values; data are shown as mean ± SD. Box-and-whisker plots, with whiskers indicating minimum/maximum, boxes indicating 25th to 75th percentile, and horizontal line indicating the median. ***P<0.001, **P<0.01, *P<0.05.

## Version 1 05/19/2026

In-Press Preview

## Version 2 06/22/2026

Electronic publication
