# Supplementary material for: Dynamic remodeling of portal vessels by Clec4g+ endothelial cells in liver growth and homeostasis
Source: JCI Insight. 2026 May 19;11(12):e201391. doi: 10.1172/jci.insight.201391 (PMC13313477; doi:10.1172/jci.insight.201391)
Supplement: Supplemental data [file jciinsight-11-201391-s050.pdf]

SUPPLEMENTAL MATERIALS and METHODS

Mice

Our study used both male and female mice, which were maintained under specific pathogen-free conditions at the Yale Animal Resources Center. The *Clec4g*-CreERT2 mouse was generated using CRISPR-Cas9-mediated genome editing. Briefly, an IRES-CreERT2 cassette was inserted immediately downstream of the stop codon at the endogenous *Clec4g* locus via pronuclear microinjection into C57Bl/6 mouse embryos. A total of 260 embryos were injected and implanted into nine foster mice, resulting in two founder mice carrying intact IRES-CreERT2 insertion. Successful germline editing was confirmed in F1 offspring by PCR detection of both 5′ and 3′ homology arms and the internal transgene. To assess Cre activity, *Clec4g*-CreERT2$^+$ mice were crossed with Ai6(RCL-ZsGreen) Cre reporter mice (1) (purchased from The Jackson Laboratory, strain #007906). To induce reporter expression, *Clec4g*-CreERT2$^+$ x ZsGreen-Ai6$^+$ mice of both sexes were injected intraperitoneally (i.p.) with tamoxifen (75 μg/g body weight) daily for five consecutive days. Mice heterozygous for both *Clec4g*-CreERT2 and ZsGreen-Ai6 alleles were used for experiments. The *Clec4g*-CreERT2$^+$ mouse line generated in this study is available to the research community upon reasonable request. Requests should be directed to the corresponding author.

Study Approval

All mice were used according to a protocol approved by the Yale University Institutional Animal Care and Use Committee.

Sex as a biological variable

Our study included male and female mice.

Liver Endothelial Cell Isolation and Flow Cytometry Analysis

Nonparenchymal liver cells were isolated as described previously (2,3). The following antibodies were then used for staining: CD31-BV605 (rat monoclonal, BioLegend, Cat. 102427) and CD45-APC/Cy7 (rat monoclonal, BioLegend, Cat. 103116). Flow cytometry was performed using an LSRII or BD Symphony, and data were analyzed with FlowJo.

Tissue collection, processing, and imaging

Liver lobes designated for imaging were resected immediately after flushing with Hanks A medium (120 mM NaCl; 5 mM KCl; 0.4 mM KH_2PO_4; 0.2 mM Na_2HPO_4; 25 mM $NaHCO_3$; 0.5 mM EGTA; 0.1% D-glucose) and prior to collagenase perfusion of the remaining lobes for flow cytometry analysis (as described above). The heart, spleen, kidneys, brain, lungs, and bones were collected immediately thereafter. For immunofluorescence staining, tissues were fixed overnight in 4% paraformaldehyde prepared in 1× TBS at 4 °C with gentle agitation. Tissues were then cryoprotected sequentially in 15% and 20% sucrose (w/v in 1× TBS), each for 24 hours at 4 °C, embedded in OCT compound, frozen on dry ice, and stored at −80°C. Sections (7 µm) were cut on a cryostat at −20°C and mounted on Unifrost Plus microscope slides (Azer Scientific). For staining, sections were quenched in ammonium chloride (0.8 g/30 mL in 1× TBS) for 10 min, rehydrated in 1× PBS for 15 min, and blocked for 1 hour at room temperature in humidified chambers with blocking buffer (1× PBS containing 0.3% Triton X-100, 1% FBS, 1% BSA, and BD Perm/Wash buffer). Slides were incubated overnight at 4 °C with primary antibodies diluted 1:100 in blocking buffer: E-cadherin (rat monoclonal, clone ECCD-2; Thermo Fisher Scientific, Cat. 13-1900), CYP2E1 (rabbit polyclonal; Abcam, Cat. ab28146), α-smooth muscle actin (rabbit polyclonal; Abcam, Cat. ab5694), LYVE-1-PE (rat monoclonal, clone ALY7; eBioscience, Cat. 12-0443-80), CD34 (rabbit monoclonal, clone EP373Y; Abcam, Cat. ab81289), and CD31 (rat monoclonal, clone MEC13.3; BD Pharmingen, Cat. 550274). After washing in 0.3% Triton X-100

in 1× PBS for 15 min, sections were incubated for 1.5 h at room temperature in humidified, light-protected chambers with secondary antibodies (1:500 in blocking buffer): goat anti-rabbit IgG (H+L), Alexa Fluor Plus 555 (Thermo Fisher Scientific, Cat. A32732), or goat anti-rat IgG (H+L), Alexa Fluor 647 (Thermo Fisher Scientific, Cat. A21247). Sections were washed and mounted with ProLong™ Gold Antifade Mountant with DAPI (Invitrogen). Confocal imaging was performed using a Leica STELLARIS 8 DIVE multiphoton confocal microscope.

Imaging Quantification

Two-dimensional single and tiled immunofluorescence images were analyzed using Fiji (Fiji Is Just ImageJ, version 2.16.0/1.54p). Portal and central vein linings were manually outlined using the Region of Interest (ROI) tool, and 4 µm-thick bands were generated along the vessel walls, excluding vessels that were structurally compromised. ZsGreen fluorescence within each ROI was binarized using the Huang thresholding algorithm (4), applied individually to each image to remove background noise while preserving the biological signal. The area and percentage of positive signal (ZsGreen$^+$ Area%) within each ROI were measured using the ROI Manager's "Measure" function. Portal veins were identified based on their proximity to E-cadherin–stained bile ducts, whereas central veins were distinguished by surrounding Zone 3 hepatocytes marked by CYP2E1 staining. Portal veins were categorized by cross-sectional area at weaning or adulthood into small (≤25th percentile), intermediate (25th–75th percentile), and large (≥75th percentile) vessels, with cutoffs of 572.1 µm² and 1248 µm² for 3-week-injected mice, and 653.6 µm² and 1456 µm² for 8-week-injected mice. DAPI$^+$ nuclei were quantified within ZsGreen$^+$ regions corresponding to the EC lining of central veins and portal veins at each time point. A minimum of three tiles from three independent mice were analyzed per time point.

Single Cell RNA-sequencing Analysis

Single-cell RNA-Seq data from healthy human livers (5) as well as mouse liver endothelial cells (2) were obtained from prior published datasets. For mouse single-cell data, only the control mouse cells were used for analysis. Seurat v. 4.3.0 and R v. 4.2.3 were used to analyze data and generate the violin plots.

Statistical analysis

Statistical analyses were performed using GraphPad Prism 10.0. Data are presented as mean ± SD for normally distributed variables and median (IQR) for non-normally distributed variables, with normality assessed by the Shapiro-Wilk test. For comparisons involving more than two groups, the Kruskal-Wallis test, followed by Dunn's multiple comparisons test, was applied. For analyses involving multiple groups across different time points or factors, two-way ANOVA with Tukey's multiple comparisons test was employed. Each experimental group included a minimum of three biological replicates. Data are shown as mean ± SD, and $p < 0.05$ was considered statistically significant.

Data availability

Values for all data points in graphs are reported in the Supporting Data Values file.

AUTHOR CONTRIBUTIONS

S.P., N.M., J.B., C.T., K.S., and S.V. performed experiments and/or analyzed data. S.P., N.M. and S.V. design experiments. S.V. supervised and funded this study. S.P., N.M., and S.V. wrote the first draft. Authorship order among the co–first authors S.P. and N.M. was determined based on contribution to experiments and manuscript preparation. All authors critically read, contributed and agree with the final version of the manuscript.

ACKNOWLEDGMENTS

We thank the Yale Center for Genome Editing, including Dr. Timothy Nottoli, Dr. William Phillbrick and their team for technical assistance with construct and mouse generation; Kathy Harris from the Yale Liver Center for technical assistance with liver perfusions; and Brandon Juarez-Ramos for technical assistance with mouse genotyping. Figure 1A was created using Biorender.com.

SUPPLEMENTAL FIGURES LEGENDS

Figure S1. *Clec4g*-CreERT2 mouse model enables selective labeling of LSECs.

(**A**) scRNA-Seq expression of *Clec4g* in human and mouse liver cell populations. (**B**) Confocal image of *Clec4g*-CreERT2^{+} mouse liver 7 days after tamoxifen administration showing ZsGreen^{+} cells throughout sinusoids with rare events (yellow arrows) within the macrovascular vessels (scale bar, 100 µm). (**C**) Confocal images of spleen, lung, heart, brain, kidney, and bone 7 days after tamoxifen administration; a few ZsGreen^{+} cells observed only in spleen (yellow arrows) (scale bar, 50 µm). ECs, endothelial cells; HSCs, hepatic stellate cells; LSECs, liver sinusoidal endothelial cells.

Figure S2. Progressive expansion of Clec4g^{+} endothelial cells into portal vessels following lineage tracing in adulthood (8-week-old).

(**A**) Representative confocal images at 7 days, 1 month, and 3 months post-tamoxifen administration in adulthood (Scale bars, 200 µm; 100 µm in insets). Portal veins (red arrows) and central veins (yellow arrows). (**B**) Quantification of ZsGreen^{+} area in central and portal veins over time (Kruskal-Wallis test); n = 3 mice at 7 days, n = 6 mice at 1 month, and n = 6 mice at 3 months post-tamoxifen administration. (**C**) Quantification of ZsGreen^{+} area in portal veins stratified by size (small, intermediate, large) at the indicated time points (Two-way ANOVA); n = 3 mice at 7 days, n = 6 mice at 1 month, and n = 6 mice at 3 months post-tamoxifen administration. (**D**) Representative confocal images of large portal veins (red arrows) branching into terminal venules (white arrows) showing progressive accumulation of labeled Clec4g^{+}ZsGreen^{+} cells over time (Scale bars, 200 µm). (**E**) Quantification of DAPI^{+} nuclei within ZsGreen^{+} regions of CVs and PVs over time (Kruskal-Wallis test). Box-and-whisker plots; whiskers = minimum/maximum, box = 25th to 75th percentile, horizontal line = median. ***$P < 0.001$, **$P < 0.01$, *$P < 0.05$; ns, not significant.

Figure S1

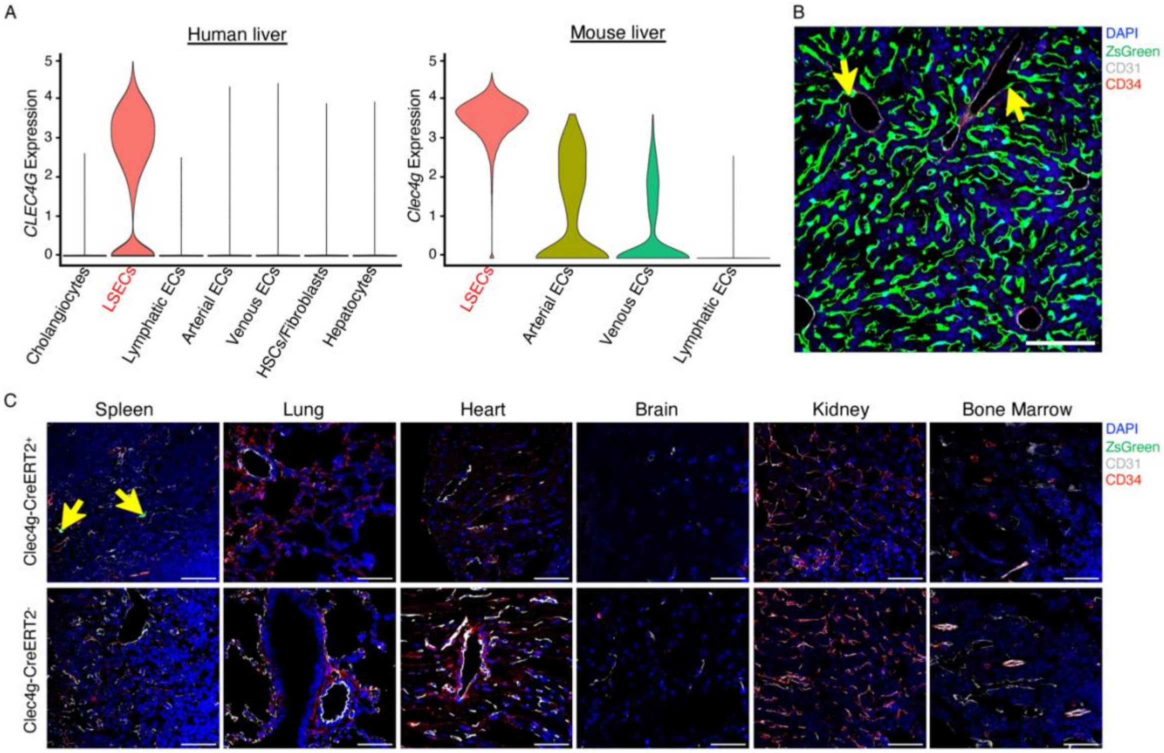
A
Human liver
CLEC4G Expression
Cholangiocytes
LSECs
Lymphatic ECs
Arterial ECs
Venous ECs
HSCs/Fibroblasts
Hepatocytes
Mouse liver
Clec4g Expression
LSECs
Arterial ECs
Venous ECs
Lymphatic ECs
B
DAPI
ZsGreen
CD31
CD34
C
Spleen
Lung
Heart
Brain
Kidney
Bone Marrow
Clec4g-CreERT2+
Clec4g-CreERT2-
DAPI
ZsGreen
CD31
CD34

Figure S2

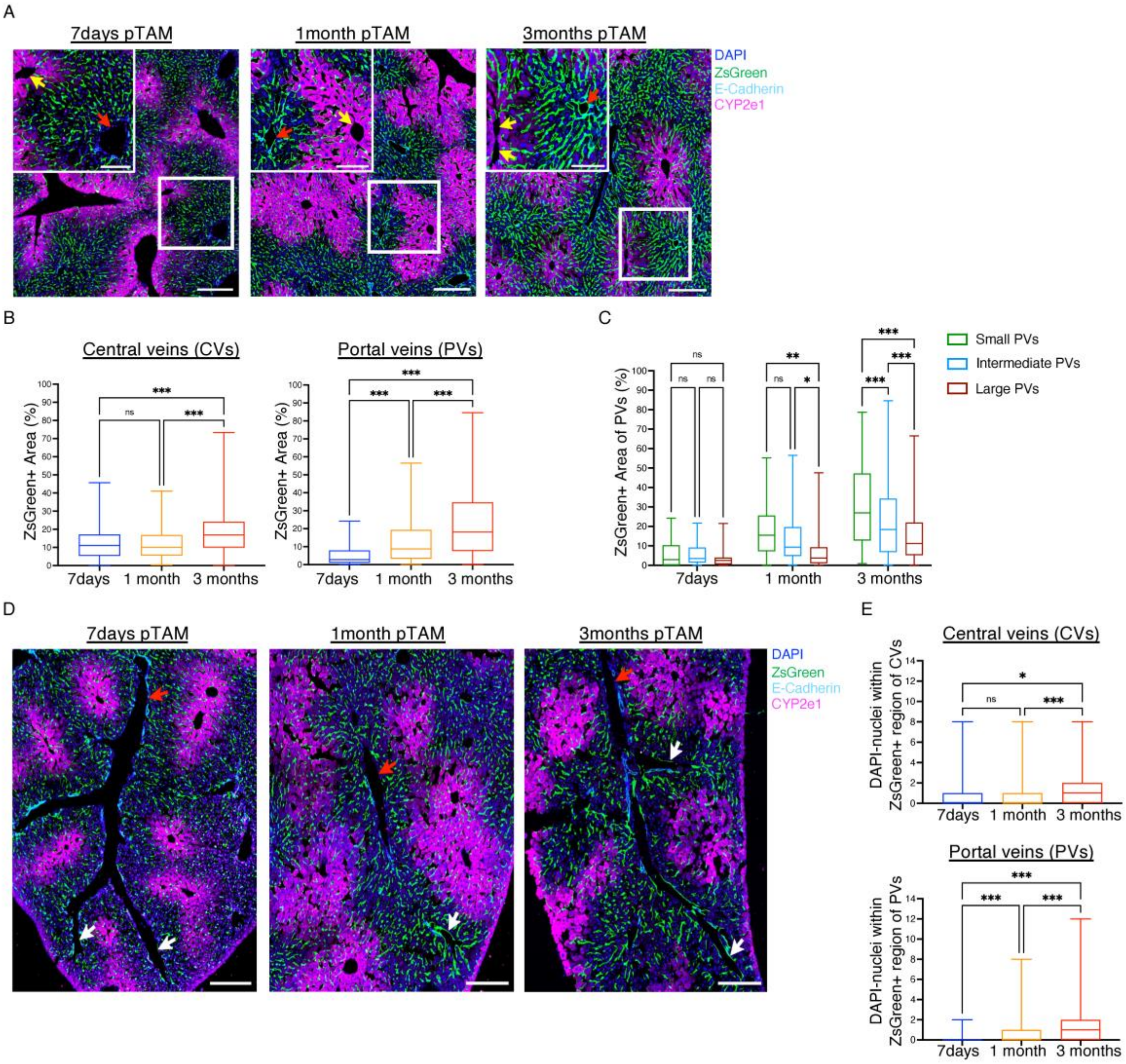
A
7days pTAM
1month pTAM
3months pTAM
DAPI
ZsGreen
E-Cadherin
CYP2e1
B
Central veins (CVs)
Portal veins (PVs)
ZsGreen+ Area (%)
7days
1 month
3 months
C
ZsGreen+ Area of PVs (%)
Small PVs
Intermediate PVs
Large PVs
D
7days pTAM
1month pTAM
3months pTAM
DAPI
ZsGreen
E-Cadherin
CYP2e1
E
Central veins (CVs)
DAPI-nuclei within ZsGreen+ region of CVs
Portal veins (PVs)
DAPI-nuclei within ZsGreen+ region of PVs
7days
1 month
3 months

REFERENCES

1. Madisen L, et al. A robust and high-throughput Cre reporting and characterization system for the whole mouse brain. *Nat Neurosci*. 2010;13(1):133-140.
2. Drzewiecki K, et al. GIMAP5 maintains liver endothelial cell homeostasis and prevents portal hypertension. *J Exp Med*. 2021;218(7):e20201745.
3. Chowdhury S, et al. Protocol for enrichment, purification, and cytocentrifugation of mouse liver endothelial cells. *STAR Protoc*. 2023;4(3):102480.
4. Huang L-K, Wang M-JJ. Image thresholding by minimizing the measures of fuzziness. *Pattern Recognit*. 1995;28(1):41-51.
5. Brancale J, Vilarinho S. A single cell gene expression atlas of 28 human livers. *J Hepatol*. 2021;75(1):219.
